# Clinical Outcome of Remnant‐Preserving and I.D.E.A.L. Femoral Tunnel Technique for Anterior Cruciate Ligament Reconstruction

**DOI:** 10.1111/os.12791

**Published:** 2020-09-25

**Authors:** Chao Su, Shi‐da Kuang, Wei‐jie Liu, Yu‐sheng Li, Yi‐lin Xiong, Xin Zhao, Shu‐guang Gao

**Affiliations:** ^1^ Department of Orthopaedics, Xiangya Hospital Central South University Changsha China; ^2^ Hunan Key Laboratory of Joint Degeneration and Injury Changsha China; ^3^ Hunan Engineering Research Center of Osteoarthritis Changsha China; ^4^ National Clinical Research Center of Geriatric Disorders, Xiangya Hospital Central South University Changsha China

**Keywords:** Anterior cruciate ligament, Arthroscopy, Femoral tunnel, Knee, Remnant preservation

## Abstract

**Objective:**

To assess the clinical results of the remnant‐preserving and I.D.E.A.L. femoral tunnel technique in the arthroscopic treatment of anterior cruciate ligament (ACL) injuries.

**Methods:**

This was a retrospective single‐center, single‐surgeon study reviewing data from November 2016 to March 2019. Based on our inclusion/exclusion criteria, a total of 31 patients (18 males, 13 females; mean age, 23.6 years) who underwent arthroscopic ACL reconstruction with the remnant preservation and I.D.E.A.L. femoral tunnel technique were recruited and had a minimum follow‐up of 12 months. Clinical data and status of knee stability were recorded. The International Knee Documentation Committee (IKDC) score, Lysholm score, and Tegner activity scale were collected both preoperatively and at a minimum of 1‐year follow‐up.

**Results:**

Statistically significant differences were detected between the preoperative and postoperative values for Lachman test and pivot‐shift test (*P* < 0.01). The mean postoperative Lysholm score was 89.6 ± 9.4, whereas the mean preoperative Lysholm score was 47.3 ± 12.8 (*P* < 0.01). The mean Tegner activity score was significantly higher at postoperative evaluation than at preoperative evaluation (6.5 ± 2.1 *vs* 2.6 ± 1.8; *P* < 0.01). The mean IKDC score was significantly improved from 49.5 ± 10.6 preoperatively to 88.2 ± 10.7 postoperatively (*P* < 0.01). No case of infection was reported. No radiograph showed any joint space narrowing or degenerative change at the last postsurgical follow‐up.

**Conclusion:**

The anatomical remnant‐preserving and I.D.E.A.L. femoral tunnel technique achieves a satisfactory clinical outcome and provides an effective option for the treatment of ACL injuries.

## Graphical abstract

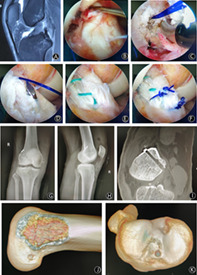

(A) MRI of the right knee showed rupture of the femoral end of ACL. (B) A routine arthroscopic examination was performed using a probe with a 30° oblique arthroscope in the anterolateral (AL) portal and another probe in the anteromedial (AM) portal. ACL avulsion from femoral insertion was observed. (C) Position of the I.D.E.A.L. femoral tunnel (arthroscopic view). A guide PDS was placed through the femoral bone tunnel. (D) The tibial tunnel (the position of the Kirschner wire) was located at the center of ACL remnant. (E) The position of ACL graft in the joint after fixation through the tibial and femoral tunnels. (F) The ACL graft and ACL remnant were sutured together with PDS suture. (G,H) Postoperative anteroposterior and lateral plain radiographs showed the correct position of the titanium plate. (I) Volumetrically rendered image from sagittal CT taken after primary ACLR showed that the tunnels were located at the anatomic position. (J) 3D CT scan reconstruction with a femoral tunnel position. (K) 3D CT scan reconstruction with a tibial tunnel position.

## Introduction

Anterior cruciate ligament (ACL) injury is one of the most common knee injuries; as estimated, there are over 300,000 ACL injuries in the United States^1^. ACL reconstruction (ACLR) is one of the most commonly‐used orthopaedic procedures that can help patients return to their former activities with the support of postoperative rehabilitation, and it is reported that about 130,000 ACLR procedures are operated each year in the United States^1,2^. Although satisfactory results have been published for the current ACLR procedures, a review article reported poor outcomes and symptomatic instability in a considerable subset of patients^3^. Several new approaches have been proposed to further stabilize and improve the postoperative results of this technique^3‐6^. One approach for improving the results of ACLR is the remnant‐preserving ACLR, which is featured with the potential advantage of promoting faster graft revascularization and maturation to achieve better knee stability and clinical outcomes. Although positive results regarding this approach have been reported by multiple clinical studies, there are also researchers stating that no difference between remnant preservation and standard ACLR was observed in postoperative knee stability and clinical scores^7^, ^8^. Another approach for improving the results of ACLR is the correct placement of femoral tunnel. Anatomical, histologic, isometric, biomechanical, and clinical data accumulated from more than 4 decades collectively points to an optimal position for the femoral tunnel within the femoral footprint^9^. This position can be summarized by the acronym I.D.E.A.L., which means the ACL graft is Isometric, in the Direct fibers, Equidistant and Eccentric, Anatomic, and Low in tension^9^. Despite being introduced in an article published as early as 5 years ago, the I.D.E.A.L. femoral tunnel technique has not been widely adopted yet. This is possibly because the clinical results of this technique have not been reported.

The aim of this study was to evaluate the clinical results of the remnant‐preserving and I.D.E.A.L. femoral tunnel technique in ACLR, based on the hypothesis that the remnant‐preserving ACLR with the I.D.E.A.L. femoral tunnel technique is an effective procedure.

## Methods

### *Inclusion and Exclusion Criteria*

The inclusion criterion were: (i) patients had subjective instability and functional impairment confirmed by a positive Lachman test and/or pivot‐shift test result; (ii) patients had ACL lesions confirmed by magnetic resonance imaging; (iii) patients had closed femoral and tibial diaphyses; (iv) patients had no history of surgery on either knee; (v) patients had no or minimal osteochondral degeneration on radiographic examination; (vi) patients who were scheduled to undergo single‐bundle ACLR using the remnant preservation and I.D.E.A.L. femoral tunnel technique with a hamstring autograft; (vii) patients had completed a clinical follow‐up of at least 12 months.

The exclusion criteria were: (i) patients without identifiable ligament tissue remaining; (ii) patients aged >50 years; (iii) the affected knee suffered from moderate to severe arthritis; (iv) patients who had previous knee surgery, rheumatological disorders, and associated malalignment (severe valgus >7° or varus knee deviation >10°); (v) patients with combined ligament injuries.

### *Patient Information*

This retrospective study was carried out upon receiving approval from our institution's ethical review board. We included clinical outcomes of 31 patients who underwent the arthroscopic ACLR with the remnant preservation and I.D.E.A.L. femoral tunnel technique alongside a clinical review between November 2016 and March 2019 (Table 1). The anterior drawer and Lachman tests were performed both preoperatively and at the last follow‐up. All operations were performed by the same surgeon.

**TABLE 1:** Characteristics of patients (*n* = 31)

Male/female sex, *n* (% male)	18/13 (58.1)
Age, mean ± SD (range), years	23.6 ± 9.6 (18–47)
Laterality (right)	19 (61.3)
Body mass index, mean ± SD (range), kg/m^2^	24.3 ± 4.0 (18.5–30.5)
Time from injury to surgery, mean ± SD (range), weeks	12.6 ± 16.7 (1–96)
Concomitant procedures, *n* (%)	
Meniscal repair	5 (16.1)
Meniscectomy	9 (29.0)
Surgical duration(min)	62 (40–90)
Follow‐up (months)	18.2 (12–36)

### *Surgical Technique*

#### *Anesthesia and Exposure*

General anesthesia was executed while the patient was placed in the supine position, with the affected side hanging down to the knee joint level. A thigh tourniquet was typically used to control bleeding and improve visualization. A routine arthroscopic examination was performed using a probe with a 30° oblique arthroscope at the anterolateral (AL) portal and a probe in the anteromedial (AM) portal. The synovial membrane, blot clots, and part of the infrapatellar fat pad were removed at 90° knee flexion.

#### *Graft Harvest and Preparation*

The tendons were harvested through a 3‐cm skin incision over the upper medial tibial metaphysis. The semitendinosus and gracilis tendons were harvested using the tendon stripper. A five‐strand graft would be prepared if the tendon had a minimum length as follows: 24 cm for the semitendinosus tendon and 16 cm for the gracilis tendon. This was required to prepare a five‐strand hamstring graft with a minimum length of 80 mm, given the need to triple the semitendinosus tendon and double the gracilis tendon. This allowed for at least 25 mm of the graft in the femoral tunnel, 30 mm inside the joint, and at least 25 mm in the tibial tunnel. Usually an 8‐mm (range, 7–9 mm) tunnel would allow passage of the graft.

#### *Femoral Tunnel Preparation*

**Fig. 1:**
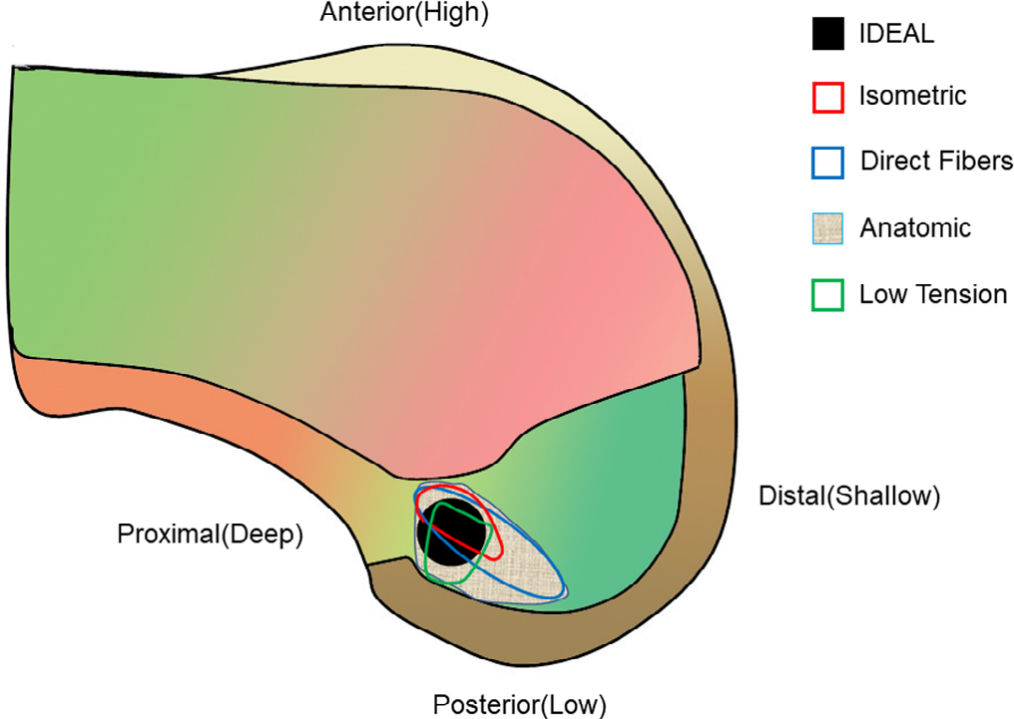
Black circle (I.D.E.A.L. femoral tunnel) locates the ideal placement of femoral tunnel in the single‐bundle anterior cruciate ligament (ACL) reconstruction. This position can be summarized by the acronym I.D.E.A.L., which refers to placing a femoral tunnel in a position that reproduces the Isometry of the native ACL, that covers the fibers of the Direct insertion histologically, that is Eccentrically located in the anterior (high) and proximal (deep) region of the footprint, that is Anatomical (within the footprint), and that replicates the Low tension‐flexion pattern of the native ACL throughout the range of flexion and extension.

After checking the femoral‐side footprint by arthroscopy (through the AL portal), the footprint center was marked with an electrocautery device passed through the AM portal. This position can be described by the acronym I.D.E.A.L., which refers to placing a femoral tunnel in a position that reproduces the Isometry of the native ACL, that covers the fibers of the Direct insertion histologically, that is Eccentrically located in the anterior (high) and proximal (deep) region of the footprint, that is Anatomical (within the footprint), and that replicates the Low tension‐flexion pattern of the native ACL throughout the range of flexion and extension (Fig. 1).

**Fig. 2:**
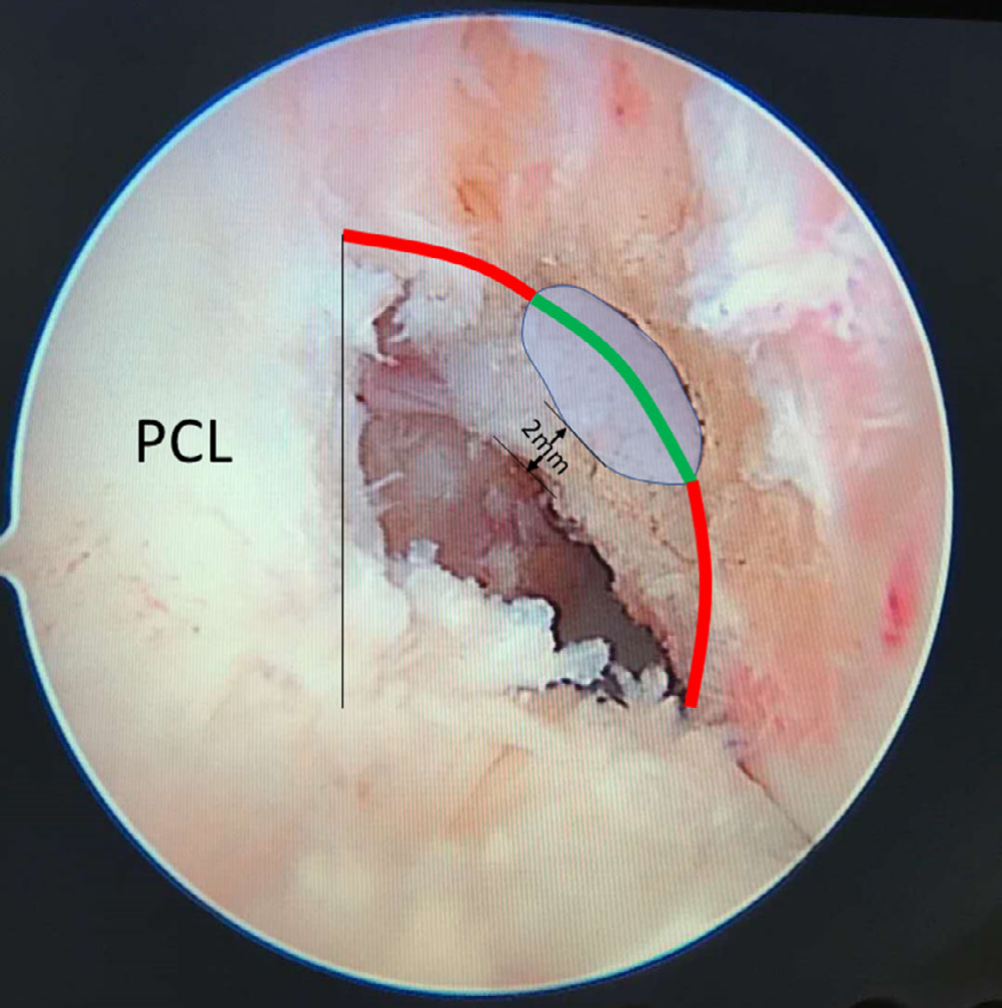
The intraoperative check for correct positioning of the femoral tunnel is to place the guide wire equidistant from the top to the bottom of the notch within the green zone and far enough posterior to position the femoral tunnel with a ≤2 mm tunnel backwall.

The intraoperative check for correct positioning of the femoral tunnel is to place the guide wire equidistant from the top to the bottom of the notch within the green zone and far enough posterior to position the femoral tunnel with a ≤2 mm tunnel backwall^10^ (Fig. 2). The femoral tunnel in single‐bundle ACLR was drilled through the AM portal using a 6 mm Smith & Nephew offset femoral aimer. The knee was bent to 90° in order to place the guide 6 mm anterior to the posterior edge of the intercondylar notch. Once the guide was in place, the knee was slowly flexed to 120° or more as permissible to ensure a more anterior directed tunnel for avoiding posterior blowout, and to provide an adequate tunnel length and safe exit for the guidewire on the lateral aspect of the thigh. A 2.4‐mm drill tip guide wire was advanced through the offset femoral guide and drilled through the femur until the guide wire “broke” through the lateral femoral cortex. A 4.5‐mm drill was advanced over the passing pin to break the lateral femoral cortex. An endoscopic cannulated drill bit that matched the graft diameter (7–9 mm) was selected and used to produce the femoral socket with a 20–25 mm depth. The femoral socket was placed as close to the posterior aspect of the notch as possible, making sure that 1–2 mm of the bone would remain as a posterior wall for the femoral socket, which indicates the I.D.E.A.L. placement (Fig. 2).

#### *Tibial Tunnel Preparation*

**Fig. 3:**
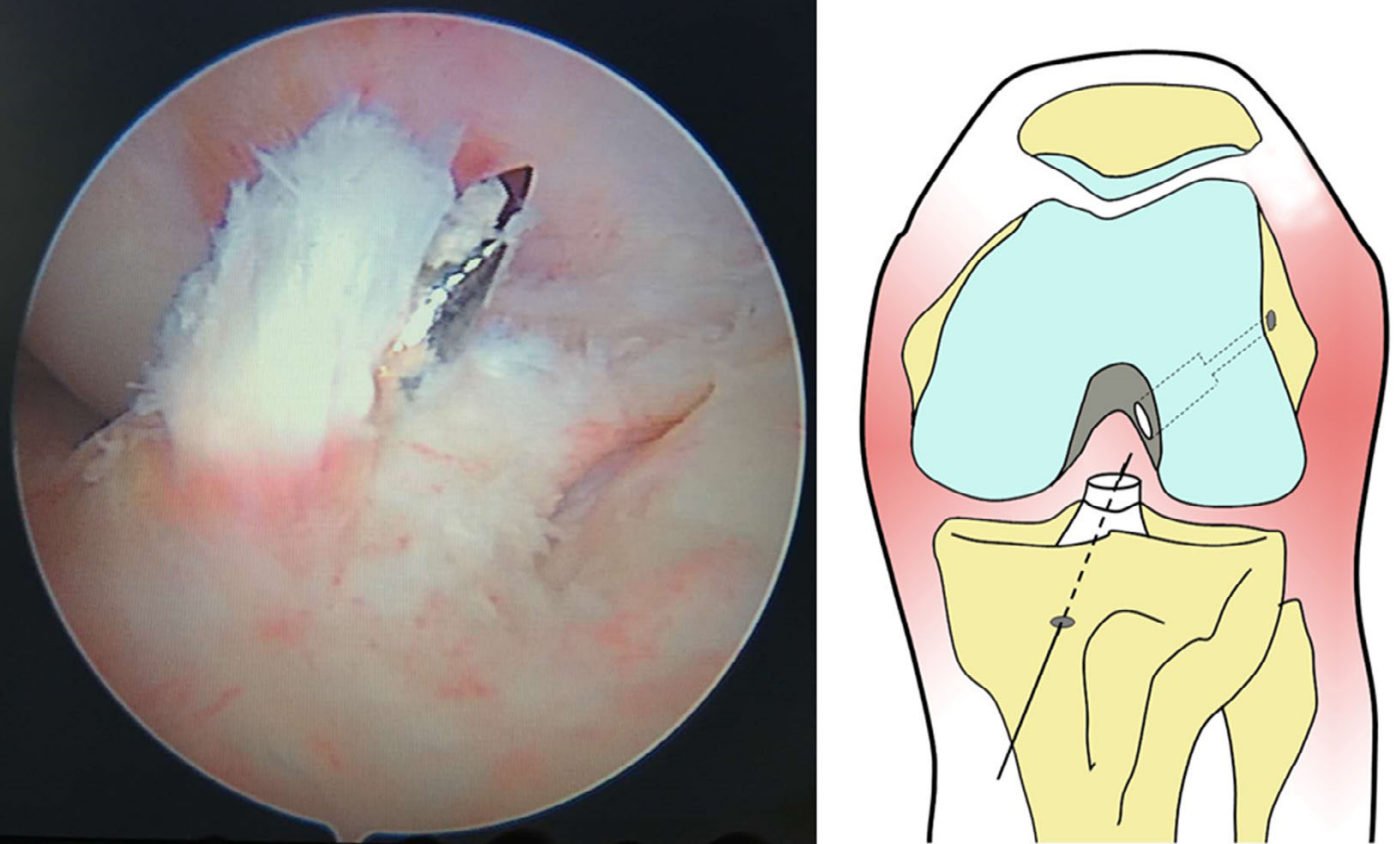
The guide pin passes through the center of the tibial remnant.

A tibial drill guide was inserted through a standard anteromedial portal and positioned in the center of the remaining ACL footprint (Fig. 3). The guidewire was advanced into the joint and carefully directed to be in line with the ACL remnant. When the optimal position was achieved, the guide pin was then over‐drilled using a cannulated reamer of 7‐mm, 8‐mm, or 9‐mm diameter to make a tibial tunnel of the same diameter as the graft. The reamer must be advanced cautiously to minimize the chance of injury to the residual remnant at the intra‐articular margin of the tibial tunnel. Penetration should stop at the base of the stump. The tunnel ends were then rounded off with an abrasion burr to reduce graft abrasion.

#### *Passage and Fixation of the Graft*

**Fig. 4:**
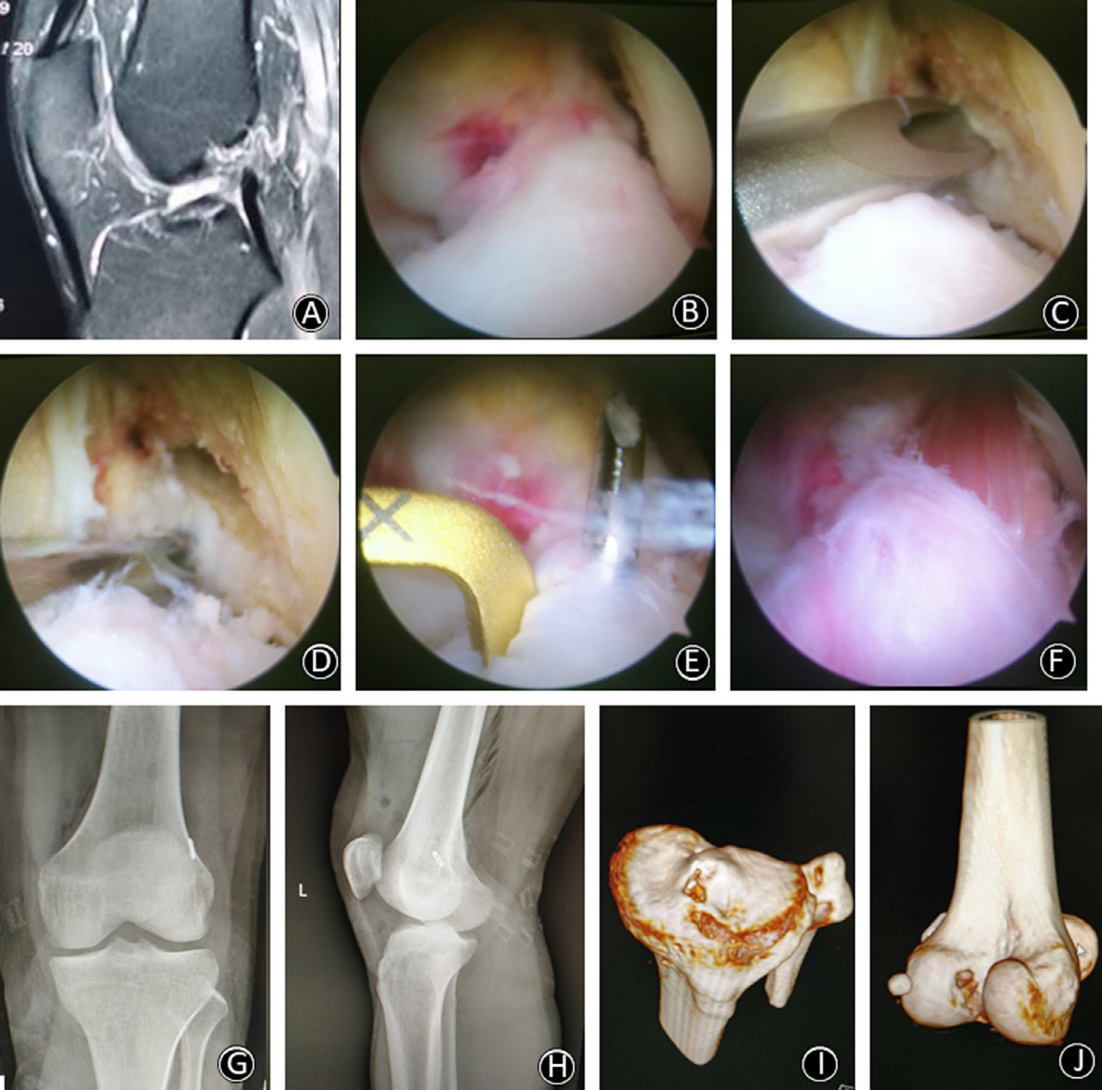
(A) MRI showed that ACL was broken and formed a low flat shape in the joint. (B) Arthroscopic rupture of ACL was observed, and the scar on posterior cruciate ligament (PCL) had healed. (C) After marking the I.D.E.A.L. point with a plasma knife, the Kirschner wire was positioned through the ACL femoral locator for femoral drilling. (D) Intraoperative check of the position of the thick femoral bone tunnel. (E) A tibial drill guide was inserted through a standard anteromedial portal and positioned at the center of the remaining ACL footprint. (F) The graft was fixed through the tibial and femoral bone tunnel in order to observe it under arthroscopy. It was found that the graft just passed the center of the ACL stump and the surface was covered by the fibers of ACL remnant. (G,H) Postoperative anteroposterior and lateral plain radiographs showed the correct position of the TightRope button. (I) 3D CT scan reconstruction with a tibial tunnel position. (J) 3D CT scan reconstruction with a femoral tunnel position.

**Fig. 5:**
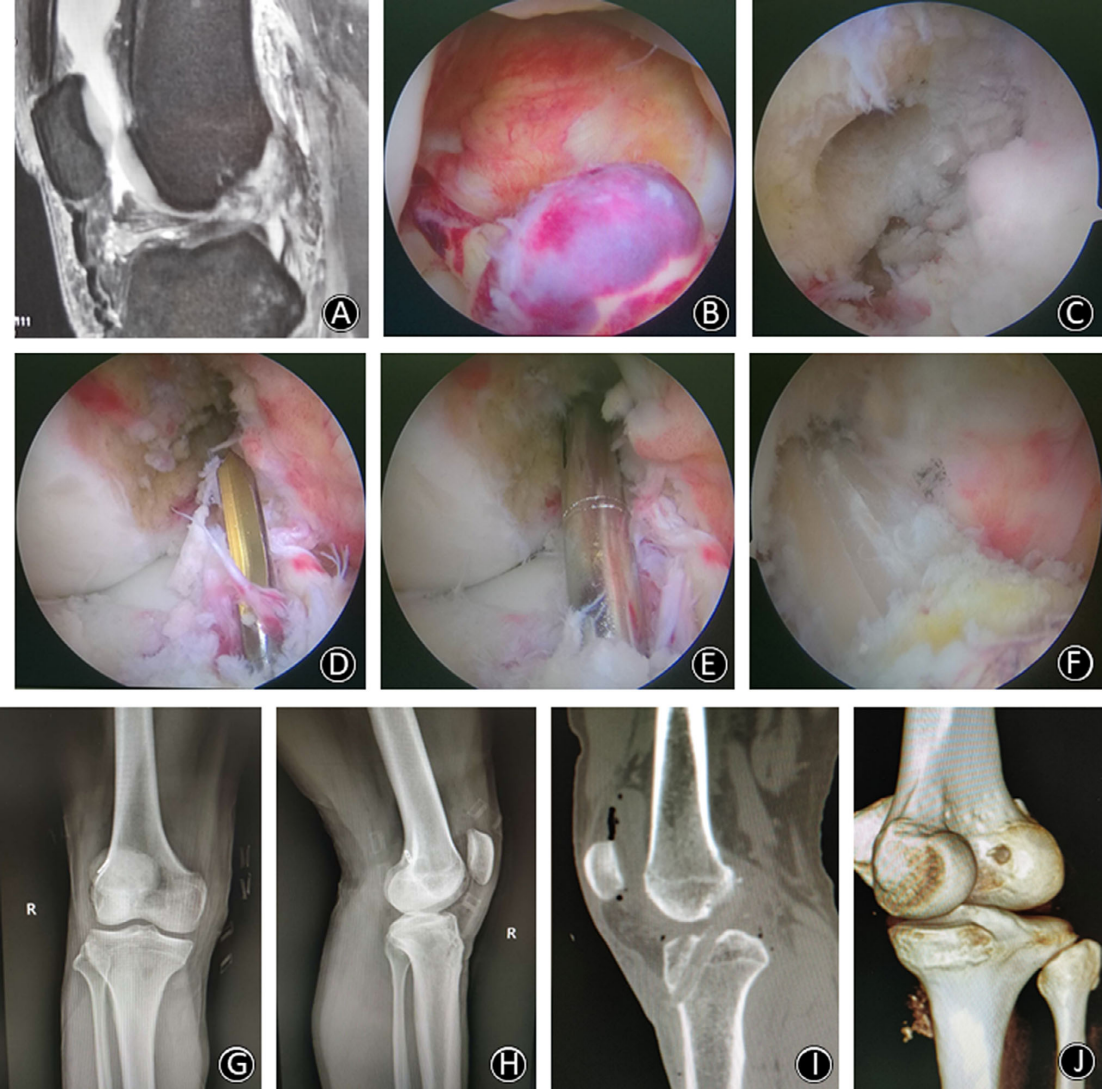
(A) MRI showed that ACL was broken, and the fiber of ACL was not continuous in the joint. (B) ACL rupture could be observed under arthroscopy, and ACL remnant showed cyclops lesion in the knee. (C) The position of the I.D.E.A.L. tunnel after the creation of the femoral tunnel. (D) The position of Kirschner wire in the tibial tunnel was located at the center of ACL remnant. (E) The shaver could reach the femoral tunnel through the tibial tunnel. It can be seen that the correspondence between the positions of the two tunnels in the joint was appropriate. (F) The graft of reconstructed ACL was well positioned in the joint. (G,H) Postoperative anteroposterior and lateral plain radiographs showed that the TightRope button was flipped on the lateral cortex of the distal femur by the distal traction applied to the graft. (I) The tibial tunnel (sagittal CT image) was located at the anatomic position. (J) 3D CT scan reconstruction with a femoral tunnel position.

**Fig. 6:**
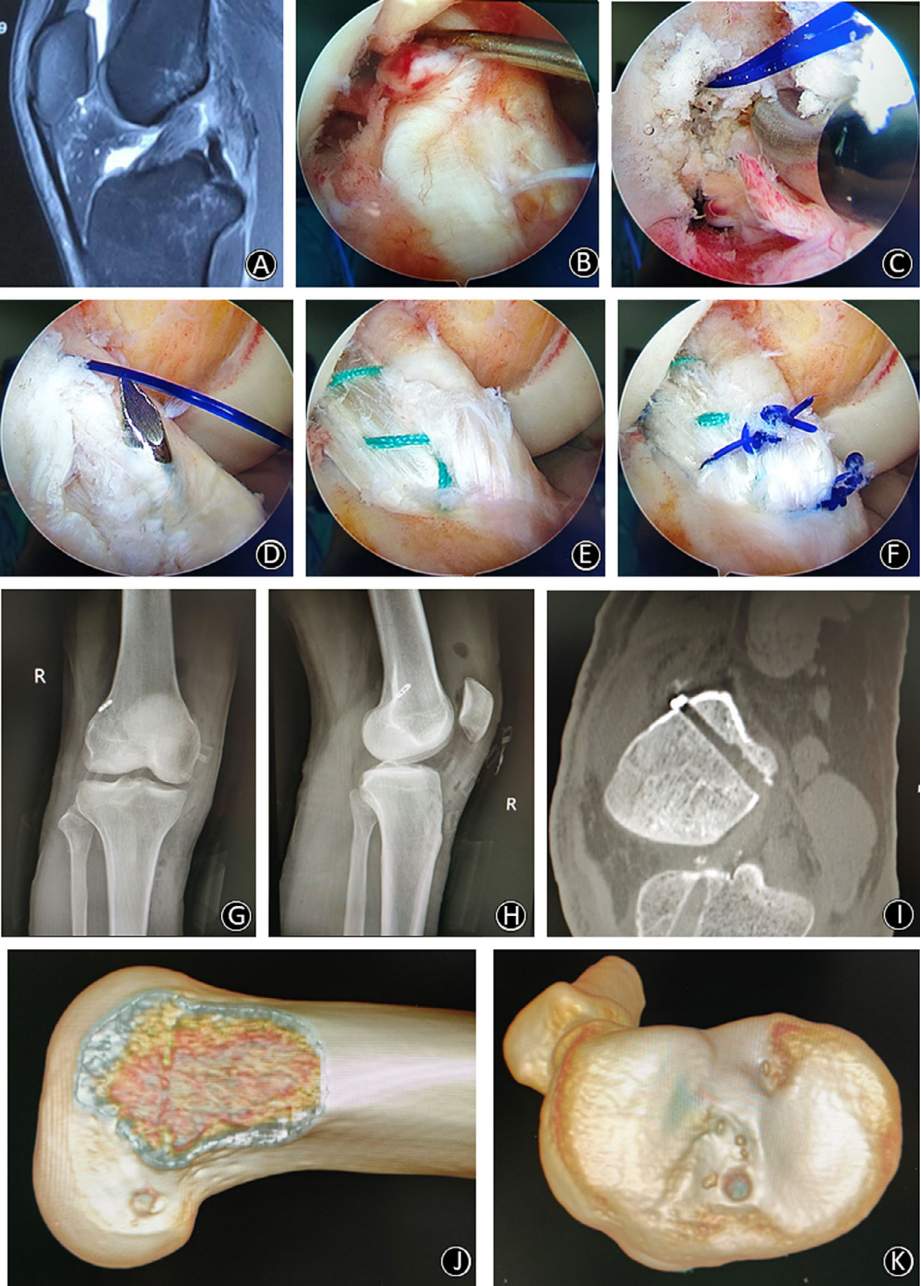
(A) MRI of the right knee showed rupture of the femoral end of ACL. (B) A routine arthroscopic examination was performed using a probe with a 30° oblique arthroscope in the anterolateral (AL) portal and another probe in the anteromedial (AM) portal. ACL avulsion from femoral insertion was observed. (C) Position of the I.D.E.A.L. femoral tunnel (arthroscopic view). A guide PDS was placed through the femoral bone tunnel. (D) The tibial tunnel (the position of the Kirschner wire) was located at the center of ACL remnant. (E) The position of ACL graft in the joint after fixation through the tibial and femoral tunnels. (F) The ACL graft and ACL remnant were sutured together with PDS suture. (G,H) Postoperative anteroposterior and lateral plain radiographs showed the correct position of the titanium plate. (I) Volumetrically rendered image from sagittal computed tomography taken after primary ACLR showed that the tunnels were located at the anatomic position. (J) 3D CT scan reconstruction with a femoral tunnel position. (K) 3D CT scan reconstruction with a tibial tunnel position.

The passing and tensioning sutures were passed through the tibial and femoral tunnel using a guide pin. The TightRope button was passed through the femoral tunnel using the passing sutures and flipped on the lateral cortex of the distal femur by the distal traction applied to the graft. The tensioning sutures were pulled slowly. Arthroscopic visualization confirmed that the previous mark on the graft was flushed with the outlet of the femoral tunnel, indicating full insertion of the graft into the femoral tunnel. While maintaining tension on the graft, the screws were inserted with the knee being flexed at 20°. The graft impingement was assessed intraoperatively with the knee in full extension. For more details of the full procedures carried out in this study, please refer to our typical cases (Figs 4, 5, 6).

### *Postoperative Management*

Quadricep exercises and straight‐leg raises were performed immediately upon completion of the operation. Full weight‐bearing was permitted immediately as tolerated. The knee was immobilized in full extension using an ACL limited‐motion brace for 1 week after the operation; then, range‐of‐motion exercises would start gradually. The 90° motion and 135° motions were allowed 4 weeks and 6 weeks after surgery, respectively. Meanwhile, at 6 weeks postoperatively, the patients would advance to the standard ACL rehabilitation protocol with the purpose to return to physical activities at 6 months. The brace was then removed 3 months after surgery. Then, at 3 and 6 months postoperatively, straight‐line running and direction changing while running, respectively, were allowed.

### *Outcome Measures*

Clinical data and status of knee stability were recorded accordingly. The International Knee Documentation Committee (IKDC) score, Lysholm score, and Tegner activity scale were collected both preoperatively and at a minimum of 1‐year follow‐up.

The IKDC evaluation form was used to detect the improvement or deterioration in symptoms, function, and sports activities. The response to each item was scored using an ordinal method. The most recent version had assigned scores for each possible response printed on the questionnaire. Scores for each item were added together to obtain a total score (excluding item 10a). The total score was calculated as (sum of items)/(maximum possible score) × 100, to give a total score of 100. An online scoring sheet was available that provides a patient's raw score and percentile score. The item regarding knee function prior to knee injury was not included in the total score. Possible score range was 0–100, where 100 = no limitation with daily or sporting activities and the absence of symptoms.

The Tegner activity scale was used to provide a standardized method of grading work and sporting activities. A score of 10 is assigned based on the level of activity that the patient selects. A score of 0 represents sick leave or disability pension because of knee problems, whereas a score of 10 corresponds to participation in national and international elite competitive sports and a score >6 can only be achieved if the person participates in recreational or competitive sport.

The Lysholm Scale is a patient‐reported outcome measure (PROM) for evaluating knee function, which consists of eight items: pain (25 points), instability (25), locking (15), swelling (10), limp (5), stair climbing (10), squatting (5), and need for support (5). The total score for a respondent is the sum of the eight items, which may range from 0 to 100. A higher score indicates a better outcome.

### *Statistical Analysis*

Statistical analyses were performed using SPSS software version 22.0 (SPSS Inc., Chicago, IL, USA). The paired *t‐*test was conducted to evaluate the differences in scores between preoperative and postoperative measurements. A *P* value <0.05 was considered as statistically significant.

## Results

### *Demographic Characteristics and Follow‐up*

Thirty‐one patients were included, consisting of 18 men (58%) and 13 women (42%). The mean age was 23.6 years (18–47 years), and the mean follow‐up was 18.2 months (12–36 months). Of the 31 patients, 14 had a concurrent meniscus tear.

There were three patients who showed extension limitation preoperatively. The medial meniscus showed a locked bucket‐handle tear in two of them, and they had 10° of extension limitation. One patient with a lateral meniscus tear also had 20° of extension limitation. A partial meniscectomy was performed in six medial and three lateral menisci, and meniscal repair was performed in three medial and two lateral menisci. Postoperatively, all of them showed full extension of the knees. The second‐look arthroscopic examination was not performed in any patients.

### *General Results*

The mean time from injury to surgery was 12.6 weeks (range, 1–96 weeks). The surgical duration was 40–90 min with a mean of 62 min, and the average blood loss was 15 mL (range, 10–20 mL). The average total duration of hospital stay was 3.2 days (range, 1 to 7 days). The average post hospital stay for the arthroscopic surgery group was 2.5 days (range, 1 to 6 days). No radiograph showed joint space narrowing or degenerative change at the last postsurgical follow‐up.

### *Ligament Laxity*

Frequencies of Lachman and pivot‐shift testing grades for preoperative and postoperative conditions are presented in Table 2. The Lachman test was negative in 29 patients, one plus in two in the postoperative conditions. The Pivot Shift test was negative in 28 patients, one plus in two and two plus in one in the postoperative conditions. Statistically significant differences were observed between the preoperative and postoperative values for Lachman test and pivot‐shift test (*P* < 0.01).

**TABLE 2:** Anterior knee stability on physical examination as reported by the subjective grading of Lachman and Pivot‐Shift maneuvers [*n*(%)]

Test	0	1	2	3
Lachman				
Preoperative	0 (0)	1 (3.2)	5 16.1)	25 (80.6)
Postoperative	29 (93.5)	2(6.5)	0 (0)	0 (0)
Pivot Shift				
Preoperative	0 (0)	0 (0)	6 (19.4)	25 (80.6)
Postoperative	28(90.3)	2 (6.5)	1 (3.2)	0 (0)

### *Lysholm Evaluation*

The mean postoperative Lysholm score was 89.6 ± 9.4 (Table 3), whereas the mean preoperative Lysholm score was 47.3 ± 12.8 (*P* < 0.01). For the general grading results, 81% were good and 19% were fair.

**TABLE 3:** Mean clinical scores (and standard deviations) on preoperative and postoperative evaluations

Parameter	Preoperative	Postoperative	*P* value
Lysholm score	47.3 ± 12.8	89.6 ± 9.4	< 0.01
Tegner scores	2.6 ± 1.8	6.5 ± 2.1	< 0.01
IKDC subjective score	49.5 ± 10.6	88.2 ± 10.7	< 0.01

### *Tegner Rating*

Preoperative and postoperative Tegner scores were used to indicate the patients' capability of returning to their pre‐injury life/work and activity level (Table 3). The mean Tegner activity score was significantly higher at the postoperative evaluation than at the preoperative evaluation (6.5 ± 2.1 *vs* 2.6 ± 1.8; *P* < 0.01).

### *IKDC* *Subjective Score*

The mean IKDC score (Table 3) was significantly improved from 49.5 ± 10.6 preoperatively to 88.2 ± 10.7 postoperatively (*P* < 0.01).

### *Complication*

One patient experienced the wound problem (fat liquefaction), which was resolved following treatment with oral antibiotics. One case of tourniquet complication was indicated by skin blisters. No case of DVT or iatrogenic neurovascular compromise was identified. No wound infection or neurovascular injury was observed.

## Discussions

Since the results of ACLR were affected by a wide range of variables, the optimal techniques are still being pursued. No previous clinical studies have been reported concerning the outcome of isolated remnant‐preserving ACLR with the I.D.E.A.L. femoral tunnel technique. Our study showed that ACLR with the remnant preservation and I.D.E.A.L. femoral tunnel technique was a good surgical option delivering satisfactory clinical results.

### *Advantages and Pearls of Remnant Preservation*

First of all, even though an ACL remnant that bridges the femur and tibia should be debrided to create the femoral and tibial tunnels in standard ACLR, it has been proven that these remnants can conserve the neuroreceptors and mechanoreceptors, which is beneficial to the joint position sense after surgery^11^, ^12^. Secondly, as far as the revascularization of the grafted tendon is concerned, an ACL remnant with abundant vascularity can exert a favorable effect allowing swifter “ligamentization” of the graft^13^, ^14^. A previous study reported that the scar pattern of the tibial stump could be classified into four types and at least 50% of the patients with ACL injury had part of the remnant tissue, even though the amount of remnant tissue might depend on the time from injury to surgery^15^. Thirdly, the preserved proprioceptive nerve fibers in the distal tibial stump would reinnervate the reconstructed ACL. Georgoulis *et al*.^16^ suggested that the presence of a proprioceptive mechanoreceptor in the remnants of the ruptured ACL might be a possible source of reinnervation of the ACL autograft. Distinguishing the effect of remnant preservation on the restoration of proprioception is difficult, and therefore, more sensitive and specific equipment or systems need to be developed to assess the proprioceptive function of the knee.

At present, the advantages of remnant preservation are difficult to demonstrate using clinical scores (e.g., Lysholm knee score, HSS score, or IKDC score) or physical examination (pivot‐shift or Lachman test). It is believed that the theoretic advantages in revascularization, ligamentization, graft incorporation, and preservation and reinnervation of mechanoreceptors can be proven by the long‐term failure rate or evaluation of proprioception. Lee *et al*.^12^ suggested that the remnant‐preserving technique in ACLR yielded better proprioceptive and functional outcomes and might help achieve better postoperative patient satisfaction. Second‐look arthroscopy is a good tool for evaluating graft healing by observing synovial coverage, graft tension, and the presence of partial tears and impingement. Kondo *et al*.^17^ reported that the arthroscopic evaluations in remnant‐preserving ACLR were significantly better than those in standard ACLR, which could severely affect the postoperative knee stability. Perhaps with a longer follow‐up, some differences would emerge in terms of re‐rupture rate, subjective results, and posttraumatic arthritis. Therefore, more randomized controlled and long‐term follow‐up studies are needed to confirm our hypothesis.

The remnant amount and the minimal injury to residual remnant are important factors in promoting the restoration of proprioceptive function. Muneta *et al*.^18^ compared the clinical outcomes of three groups (classified according to the remnant volume: ≤30%, 35%–55%, and ≥60%) and found that the remnant volume was weakly correlated with the postoperative outcome. On the other hand, Nakayama *et al*.^19^ indicated that a large remnant might increase the incidence of cyclops lesions and extension loss. In this study, the length of the stump was properly preserved during the operation; meanwhile, reaming was stopped at the base of the remnant for minimal injury to the residual remnant. Roof impingement with knee extension by scarring the graft in the anterior intercondylar area (cyclops lesion) and anterior placement of the tibial tunnel can be prevented because the tibial tunnel is positioned within the boundary of the normal ACL remnant and surrounded by the remnant tissue. The tibial remnant leaves no definite gap at the exit point of the tunnel and prevents synovial fluid from tracking along the graft. For these reasons, we believe that our technique can well prevent tibial tunnel enlargement.

### *Advantages and Pearls of I.D.E.A.L. Femoral Tunnel Technique*

Anatomic femoral tunnel placement such that the ACLR graft lies within the native ACL femoral attachment site and has been shown to better restore anterior tibial translation, rotational stability, and normal knee kinematics^20^, ^21^, ^22^. Clinical studies have demonstrated that the most common technical error resulting in instability or graft failure after ACLR is nonanatomic graft placement and that the nonanatomic femoral tunnel position is closely correlated with poorer clinical outcome scores^23^, ^24^. Previous studies have reported that the femoral socket is localized in the center of the entire footprint with a single‐bundle technique and the sockets are created in the centrums of both the AM and PL bundles with a DB technique^20^, ^21^, ^22^, ^23^, ^24^. However, in light of the discrepancy between the size of the femoral footprint and the mid‐substance of the native ACL, it seems reasonable that optimizing the position of the ACL femoral tunnel may be more complex than simply centralizing the tunnel within the footprint or attempting to maximize the footprint coverage. According to Pearle^9^, the acronym I.D.E.A.L. reminds surgeons to place the ACL graft in a femoral tunnel position that reproduces Isometry of native ACL, that covers fibers of Direct insertion, that is Eccentrically located in anterior (high) and proximal (deep) region of footprint, that is Anatomical (within femoral footprint), and that replicates Low tension‐flexion pattern of native ACL throughout the range of flexion and extension.

The intraoperative check for the correct positioning of the femoral tunnel is to place the guide wire equidistant from the top to the bottom of the notch within the green zone and far enough posterior to position the femoral tunnel with a ≤2 mm tunnel backwall^10^ (Fig. 2). Centering the femoral guide wire in the green zone has the advantages of setting the graft length isometrically, centering the graft in the direct fibers of the origin of the native ACL anatomically, and restoring low graft tension, which are associated with high function, full motion, and high stability^25^. The advantage of the green zone with the I.D.E.A.L. philosophy lies in that it enables an anterior medial or transtibial drilling technique while providing the ability to consistently place the tunnel within the green zone and allows a small amount of latitude in order to support the individuality of the patient's anatomy and notch. However, the achieved tunnel position for transtibial drillers was found to be higher in the intercondylar notch than that for other drilling techniques (outside‐in and AM portal)^26^. In this study, the use of an AM‐portal ACLR technique could initially mark the tunnel position with an electrocautery device and then verify this position by switching to the AM portal. The 6‐mm offset femoral aimer used in the operation could control the distance of the femoral tunnel to the posterior backwall.

### *Limitations*

Several limitations of the present study deserve comments. Firstly, our analysis was limited by the small number of cases and non‐comparative single‐arm reports of surgical results. More patients are expected to further validate our findings and conclusions. Data of comparison relative to the single‐bundle ACLR with remnant preservation or the standard ACLR is needed. Secondly, another drawback of this study was that the parameters of magnetic resonance imaging (MRI) or computed tomography (CT) were not evaluated. Thirdly, the mean term of follow‐up was relatively short. It is critical to include subjects with longer terms of follow‐up in order to better evaluate growth disturbances. Lastly, scientific research should be carried out to prove the benefit of the remnant preserving technique.

## Conclusions

In conclusion, our anatomical remnant‐preserving and I.D.E.A.L. femoral tunnel technique is an easy‐to‐operate and effective method for the treatment of ACL injuries, with excellent postoperative stability and clinical results.
